# Analysis of Factors Associated with the Ocular Features of Congenital Cataract Children in the Shanghai Pediatric Cataract Study

**DOI:** 10.1155/2017/8647435

**Published:** 2017-09-20

**Authors:** Wenwen He, Ting Sun, Jin Yang, Guoyou Qin, Zhenyu Wu, Xiangjia Zhu, Yi Lu

**Affiliations:** ^1^Department of Ophthalmology, Eye and Ear, Nose, and Throat Hospital of Fudan University, 83 Fenyang Road, Shanghai 200031, China; ^2^Key Laboratory of Myopia, Ministry of Health, Shanghai 200031, China; ^3^Shanghai Key Laboratory of Visual Impairment and Restoration, Shanghai 200031, China; ^4^Department of Biostatistics, School of Public Health, Key Laboratory of Public Health Safety, Ministry of Education, Fudan University, Shanghai 200032, China

## Abstract

**Purpose:**

To investigate the ocular features of children with congenital cataract in a tertiary referral eye center in East China.

**Methods:**

We retrospectively reviewed the clinical data of congenital cataract children who underwent cataract surgery between April 2009 and April 2014 at the Eye and ENT Hospital of Fudan University and identified factors associated with the axial length (AXL) and corneal curvature (*K* value).

**Results:**

We included 493 children, 210 with unilateral and 283 with bilateral cataract. The mean AXL was 22.03 ± 1.97 mm and the mean *K* value was 43.61 ± 1.86 D. Age showed a linear correlation with AXL in unilateral cataract eyes and a logarithmic correlation with AXL in bilateral cataract eyes (both *P* < 0.001). AXL was longer and the *K* value was smaller (both *P* < 0.01) in boys than in girls after adjusting for age and cataract laterality. AXL was longer in unilateral cataract eyes than in bilateral cataract eyes after adjusting for age and gender (*P* = 0.004). In children with unilateral cataract, AXL was significantly longer in the affected eye than in the contralateral eye (*P* < 0.001).

**Conclusion:**

Age, gender, and cataract laterality together contribute to the development of ocular features of congenital cataract children, especially for AXL.

## 1. Introduction

Congenital cataract is the leading cause of visual impairment worldwide according to the updated global report by the World Health Organization [1]. Cataract surgery with or without intraocular lens (IOL) implantation is still the most common treatment method for congenital cataract [2, 3]. The measurement of ocular features, especially axial length (AXL) and corneal curvature (*K* value), is essential for IOL power calculation [4]. However, the formulae used in IOL power calculation were derived from data in adults and may be inaccurate for children [5, 6]. Furthermore, although selection of the target refractive error is usually dependent on age [4, 7], other factors may influence the ocular development of children with congenital cataract that should also be considered. Thus, it is important to analyze these factors in order to choose the most suitable IOL for children with congenital cataract.

Although the AXL and *K* value in children with congenital cataract have been reported [8–10], few studies have determined these parameters in Chinese children or examined the correlation between AXL and *K* value. The Eye and Ear, Nose and Throat (ENT) Hospital of Fudan University is the largest and best tertiary referral eye center in East China and is involved in the care of nearly all children with cataract in this region. Thus, the purpose of this cross-sectional study was to assess the AXL and *K* value of children with congenital cataract who underwent cataract surgery at ≤18 years of age at our hospital in 5 years. We also examined the factors associated with the AXL and *K* value, including age, gender, and laterality, and analyzed the relationship between the AXL and *K* value. Our results should provide a reference for IOL power selection in children undergoing cataract surgery in the future.

## 2. Methods

The Institutional Review Board of the Eye and ENT Hospital of Fudan University, Shanghai, China, approved this retrospective study affiliated to the Shanghai Pediatric Cataract Study. All procedures adhered to the tenets of the Declaration of Helsinki. The Shanghai Pediatric Cataract Study was registered at ClinicalTrials.gov (accession number NCT03063216).

### 2.1. Children

We retrospectively reviewed the medical records of children with congenital cataract who underwent cataract surgery with or without IOL implantation at the Eye & ENT Hospital of Fudan University between April 2009 and April 2014. Only children aged ≤18 years who were diagnosed with congenital cataract were enrolled. Children with systemic diseases, previous trauma, lens subluxation, or other conditions likely to affect the measurement of the AXL and *K* value were excluded. Other types of childhood cataracts due to trauma, exposure to radiations, metabolic and other acquired causes were excluded. We also excluded the eyes in which AXL was not measured. Data collected included the age at surgery, gender, cataract laterality (unilateral or bilateral), and the AXL and mean *K* values of both eyes.

### 2.2. Examinations

AXL was measured using an IOLMaster® 500 (Carl Zeiss AG, Oberkochen, Germany) before surgery in older children who cooperated with the procedure. If the AXL could not be measured in conscious infants, a contact A-scan (Tomey EM-3000, Nagoya, Japan) was performed after instillation of topical anesthetic and sedation with 10% chloral hydrate before surgery or under general anesthesia at surgery. The mean *K* value was only obtained in children who cooperated during measurements with the IOLMaster. Each eye was measured 5–10 times, and the mean value was recorded. The preoperative examinations were performed only by one special technician in biometry for children.

### 2.3. Main Outcome Measures

The main outcome measures were the AXL and *K* value of children with congenital cataract. In children with bilateral cataract, we randomly selected one eye for analysis to avoid correlation effects. The children were divided into four groups for further analyses according to their age at surgery: 0-1, 1-2, 2–6, 6–12, and 12–18 years old. The associations of age, gender, and cataract laterality with the AXL and mean *K* value and the correlation between the AXL and *K* value were also determined.

### 2.4. Statistical Analysis

All statistical analyses were performed using SPSS version 11 (SPSS Inc., Chicago, IL, USA). Quantitative variables are presented as the mean ± standard deviation (SD), and categorical variables are described as the number and/or percent of children. Mean ± 3SD was set as the cut points of the quantitative variables, and data out of this range was excluded from analysis. Student's *t*-test was used to compare continuous variables, and the *χ*^2^ test was used to compare categorical data between two groups. Univariate analysis of covariance (ANCOVA) was used to adjust for age, gender, and laterality. The correlation between AXL and mean *K* value was determined using Pearson's correlation analysis, while linear or logarithmic regression was also used to assess associations between these variables and other explanatory variables. *P* values of <0.05 were considered statistically significant.

## 3. Results

### 3.1. Characteristics of the Children

**Figure 1:**
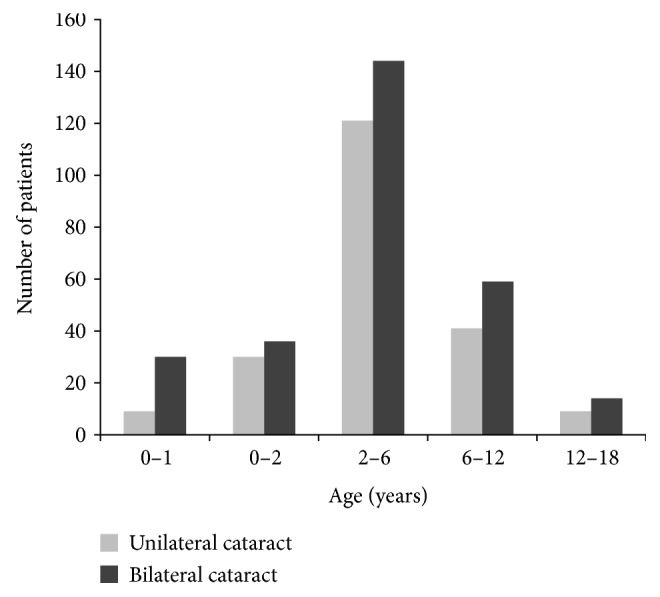
Distribution of children with congenital cataract according to the age at surgery and unilateral/bilateral cataract. There were more bilateral cases aged 0-1 year than unilateral cases (10.6% versus 4.3%, *χ*^2^ test, *P* = 0.010).

Overall, 493 children with congenital cataract satisfied our eligibility criteria and were included in this study. All of the children had complete AXL data for both eyes. There were 210 children with unilateral cataract and 283 with bilateral cataract. The mean age at surgery of all children combined was 4.72 ± 3.36 years and ranged from 0.25 to 17.40 years. The mean ages of children with unilateral and bilateral cataract were 4.93 ± 3.29 and 4.57 ± 3.40 years, respectively, and were not significantly different between these groups (Student's *t*-test, *P* = 0.250). The age distribution of children with congenital cataract is shown in Figure 1. There were more bilateral cases aged 0-1 year than unilateral cases (10.6% versus 4.3%, *χ*^2^ test, *P* = 0.010). The largest subgroup was children aged 2–6 years (53.75%). Overall, 121 boys and 89 girls had unilateral cataract while 175 boys and 108 girls had bilateral cataract. The distribution of boys and girls was not significantly different between the two groups (*χ*^2^ test, *P* = 0.320).

### 3.2. Correlations between Age, Gender, and Laterality with AXL and Mean *K* Value

**Figure 2:**
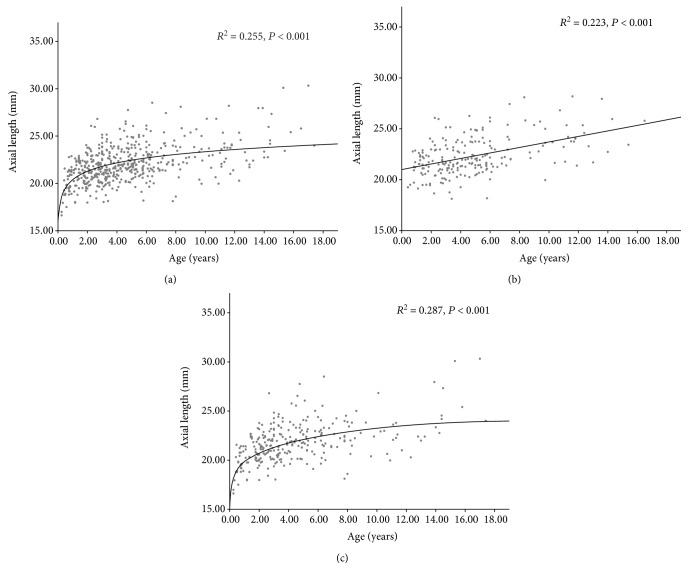
Correlations between age and the axial length of affected eyes in all children with congenital cataract ((a) logarithmic regression, *R*^2^ = 0.255, *P* < 0.001), children with unilateral cataract ((b) linear regression, *R*^2^ = 0.223, *P* < 0.001), and children with bilateral cataract eyes ((c) logarithmic regression, *R*^2^ = 0.287, *P* < 0.001).

After excluding cases with AXL out of cut points, there were 486 eyes used for further analysis. The excluded cases were all boys with bilateral cataract. The mean AXL of the 486 eyes with congenital cataract was 22.03 ± 1.97 mm and ranged from 16.61 to 30.32 mm. The logarithmic correlation between AXL and age in 486 children with congenital cataract is shown in Figure 2(a) (*R*^2^ = 0.255, *P* < 0.001). We found a significant linear correlation between age and AXL in children with unilateral cataract (Figure 2(b), *R*^2^ = 0.223, *P* < 0.001) and a significant logarithmic correlation between age and AXL in children with bilateral cataract (Figure 2(c), *R*^2^ = 0.287, *P* < 0.001).

The mean *K* value was successfully measured in 201 children aged >2 years, of which 2 cases were out of cut points, and then, data from 87 unilateral cataract and 112 bilateral cataract were used. The mean *K* value of these children was 43.61 ± 1.86 D and ranged from 38.16 to 49.75 D. However, age was not correlated with the mean *K* value in these children (linear regression, *R*^2^ < 0.001, *P* = 0.927).

As shown in Table 1, AXL was significantly longer (ANCOVA, *P* = 0.010) and the mean *K* value (ANCOVA, *P* < 0.001) was significantly smaller in boys than in girls after adjusting for age and cataract laterality. The AXL and mean *K* value were also compared between boys and girls in age groups of children (Table 1). In these analyses, we found that AXL was significantly different between boys and girls in children aged 0-1 year (ANCOVA, *P* = 0.037), children aged 2–6 years (ANCOVA, *P* = 0.036), and children aged 6–12 years (ANCOVA, *P* = 0.034). The mean *K* value was significantly larger in girls than in boys aged 2–6 years old (ANCOVA, *P* = 0.004).

The AXL and mean *K* value are compared between children with unilateral cataract and children with bilateral cataract in Table 2. After adjusting for age and gender, AXL was significantly longer in children with unilateral cataract than in children with bilateral cataract (ANCOVA, *P* = 0.004). These differences were significant in children aged 1-2 and 6–12 years (ANCOVA, *P* = 0.046 and *P* = 0.020). The mean *K* value was not significantly different between children with unilateral cataract and children with bilateral cataract after adjusting for age and gender (ANCOVA, all *P* > 0.05).

The AXL and mean *K* value of affected eyes and contralateral eyes in children with unilateral congenital cataract are shown in Table 3. In all children, the AXL was significantly longer in the affected eye than in the contralateral eye (paired *t*-test, *P* < 0.001). This difference was also observed in all age groups (paired *t*-test, all *P* < 0.05), except in children aged 12–18 years (paired *t*-test, *P* = 0.123). By contrast, the *K* values were similar between the affected eye and the contralateral eye in all children and in each age group (paired *t*-test, all *P* > 0.05). In children with bilateral congenital cataract, there were no differences in the AXL or *K* value between the randomly selected eye and the contralateral eye (Table 4, paired *t*-test, all *P* > 0.05).

### 3.3. Correlation between the AXL and *K* Value in Children with Congenital Cataract

After excluding cases with AXL and *K* value out of cut points, the *K* values were negatively correlated with AXL in all children, children with unilateral cataract, and children with bilateral cataract (Pearson's correlation analysis, *r* = −0.314, *P* < 0.001; *r* = −0.237, *P* = 0.027; and *r* = −0.373, *P* < 0.001, resp.).

## 4. Discussion

It is important to determine the ocular features in children with congenital cataract to understand the development of their affected eyes, to calculate the IOL power precisely, and to ensure that the target refractive error is correctly determined. Prior studies reported that age is significantly associated with ocular features [9, 11], as are gender and cataract laterality [9, 10]. However, most studies used univariate analyses and studied the effects of other factors in children who were only grouped by age [9, 12]. In our study, we used ANCOVA to adjust for each variable and identify which variables were independently associated with ocular features. This approach may be more reliable than the analyses used in prior reports [9, 12]. Furthermore, ocular features differed between races, but few studies have focused on Asian children [8–10]. Thus, our study could fill gaps in our knowledge, especially in terms of the ocular features of children with congenital cataract in East China. In this cross-sectional study, we analyzed the ocular characteristics of children with congenital cataract who underwent cataract surgery in our hospital at an age of ≤18 years. We found that age showed a linear correlation with AXL in children with unilateral cataract and a logarithmic correlation with AXL in children with bilateral cataract. Gender and cataract laterality were also associated with the AXL of children with congenital cataract. In children aged ≤12 years with unilateral cataract, the AXL of the affected eye was significantly longer than that of the contralateral eye. In addition, AXL showed a negative correlation with the mean *K* value in children with congenital cataract.

The mean age of the enrolled children was 4.72 ± 3.36 years, which was slightly older than the mean ages reported in previous studies [9, 12]. This may be due to the fact that children with cataract usually underwent IOL implantation after 1 year of age in our center. AXL was not available in younger children who underwent cataract surgery without IOL implantation; these children were excluded from this study and this is a limitation of our study. In addition, the operated age between unilateral and bilateral cases was not different in our study since some bilateral cases may have one-stage surgery without IOL implantation and AXL data under 1 year old. A study on baseline characteristics of children who underwent cataract surgery less than 13 years old in North America showed a mean operative age of 4.2 years, which was similar to ours, but this study has more unilateral cases (59% versus 43%) and has more cases (34%) who underwent cataract surgery at 0-1 year [13].

We found a significant logarithmic correlation between age and AXL in all children and a marked increase in AXL in children aged ≤2 years, consistent with prior studies [9, 12, 14]. Trivedi and Wilson reported that the AXL at <1 year of age was significantly different from that of the other age groups and extended rapidly in the first year of age [9]. However, the earlier studies did not separate children with unilateral or bilateral cataract when analyzing the relationship between age and AXL [12, 14]. We found a significant linear correlation (*R*^2^ = 0.223, *P* < 0.001) rather than a logarithmic correlation (*R*^2^ = 0.195, *P* < 001) between age and AXL when the analysis was limited to children with unilateral cataract. This suggests that the eyeball may develop differently between children with unilateral and bilateral cataract. This finding may help explain why the visual acuity of the affected eye in children with unilateral cataract was usually worse than that of the eyes in children with bilateral cataract in previous studies [15–17].

Previous studies revealed that AXL was longer in boys than in girls with congenital cataract [9, 12]. We confirmed this finding after adjusting for age and cataract laterality. This may be due to the positive correlation between AXL and head size reported elsewhere [18, 19]. According to the World Health Organization Child Growth Standards, at <5 years of age, boys have a larger head circumference than girls. This may explain the difference in AXL between boys and girls in children aged 0-1 year and 2–6 years.

After adjusting for age and gender, AXL was found to be longer in children with unilateral cataract than in children with bilateral cataract, especially in those aged 1 to 2 years. This is an important period of time for AXL development [20], and the AXL may increase quicker during this time in the affected eyes of children with unilateral cataract [12]. The growth of eyes affected by cataract may be influenced by two mechanisms: form deprivation may lead to a longer AXL, whereas ocular anomalies may be associated with a shorter AXL. We surmise that unilateral cataract is a unilateral anomaly, which may be due to environmental factors rather than genetic factors, and develop into form-deprivation myopia. Besides, the AXL of the affected eye was also longer than that of the contralateral eye in our study, which was opposite to the results of some studies in Western countries [9]. Racial differences may explain these differences. Myopia and high myopia are more common in Asian populations than in Western populations [21, 22]. Thus, we think that form deprivation in unilateral cataract is more likely to lead to myopia or a longer AXL in Chinese children than in other races. What is more, a recent research from the Infant Aphakia Treatment Study showed this opposite result in unilateral cataract infants aged 1 to 7 months [23]. Compared with our study, which contained most unilateral cataract children aged over 2 years, it suggested that the occurrence of form deprivation myopia may need more than one year.

In our study, we did not find a correlation between age and the mean *K* value. One possible explanation may be that the mean *K* value could only be determined in children aged >2 years. It was previously reported that the *K* value was negatively correlated with age in children with congenital cataract aged <6 months old [10]. This means that the mean *K* value decreased significantly from birth to 6 months of age. However, this correlation disappeared in children aged >6 years [10].

Consistent with earlier studies, we found that the mean *K* value was smaller in boys than in girls with congenital cataract, [10] and may be due to the difference in head circumference between boys and girls. Previous studies reported that the *K* value in the affected eye of children with unilateral cataract was greater than that of children with bilateral cataract and the contralateral eye [10]. However, we did not observe a similar phenomenon. This may be due to the fact that the largest differences were observed in children aged 0–6 months [10], an age group excluded from our study.

We also examined the correlation between the AXL and mean *K* value in children with congenital cataract and found a negative correlation between these variables. This suggests that AXL is more strongly associated with the mean *K* value than age. Therefore, surgeons should consider the age, gender, and AXL when estimating the *K* value in children who did not cooperate with keratometry.

In conclusion, we determined the ocular features of children aged ≤18 years with congenital cataract who underwent cataract surgery in our hospital over a 5-year period. Age, gender, and cataract laterality together contribute to the development of ocular features, especially for AXL. We found different correlation types between age and AXL in children with unilateral cataract and bilateral cataract, which suggest that the eyeball may develop differently between children with these two types of congenital cataract. Unlike children in Western countries, Chinese children with unilateral congenital cataract usually have longer AXL in the affected eye than the fellow eye. Gender may have more influence on mean *K* value than age in children ≥2 years old. In addition, the mean *K* value decreased with increasing AXL. Our study described the relationships between demographic characters and ocular features in Chinese congenital cataract children and may provide a reference for those who cannot cooperate with these measurements, especially in corneal curvature examination.

## Figures and Tables

**Table 1:** Comparison of axial length and mean *K* value between boys and girls after adjusting for age and the cataract laterality.

Age (years)	AXL (mm)			*K* value (D)		
	Boys	Girls	*P*	Boys	Girls	*P*
0-1	20.17 ± 1.59	19.56 ± 1.29	**0.037** ^∗^	—	—	*—*
1-2	21.03 ± 1.23	21.19 ± 1.54	0.989	—	—	—
2–6	22.16 ± 1.73	21.73 ± 1.53	**0.036** ^∗^	43.19 ± 1.69	44.11 ± 1.68	**0.004** ^∗^
6–12	23.28 ± 1.97	22.33 ± 1.80	**0.034** ^∗^	43.43 ± 1.90	44.31 ± 2.27	0.063
12–18	24.92 ± 4.22	24.58 ± 1.73	0.697	42.76 ± 1.41	44.23 ± 1.91	0.104
All children	22.23 ± 1.98	21.73 ± 1.93	**0.010** ^∗^	43.22 ± 1.73	44.19 ± 1.90	**<0.001** ^∗^

^∗^Significantly different between boys and girls after adjusting for age and cataract laterality by analysis of covariance. Results are presented as the mean ± standard deviation. AXL = axial length.

**Table 2:** Comparison of axial length and *K* value between children with unilateral or bilateral congenital cataract after adjusting for age and gender.

Age (years)	AXL (mm)			*K* value (D)		
	Unilateral	Bilateral	*P*	Unilateral	Bilateral	*P*
0-1	21.03 ± 1.33	19.54 ± 1.37	0.093	—	—	—
1-2	21.43 ± 1.20	20.86 ± 1.45	**0.046** ^∗^	—	—	—
2–6	22.11 ± 1.72	21.90 ± 1.62	0.494	43.66 ± 1.82	43.51 ± 1.69	0.763
6–12	23.48 ± 1.99	22.45 ± 1.82	**0.020** ^∗^	43.60 ± 1.57	43.95 ± 2.45	0.385
12–18	24.53 ± 1.93	24.79 ± 3.07	0.722	43.18 ± 0.43	43.21 ± 2.08	0.896
All children	22.34 ± 1.89	21.79 ± 2.01	**0.004** ^∗^	43.60 ± 1.65	43.62 ± 2.01	0.650

^∗^Significantly different between children with unilateral and bilateral cataract after adjusting for age and gender using analysis of covariance. Results are presented as the mean ± standard deviation. AXL = axial length.

**Table 3:** Comparison of axial length and *K* value between the affected eye and the contralateral eye in children with unilateral congenital cataract.

Age (years)	AXL (mm)			*K* value (D)		
	Affected eye	Fellow eye	*P*	Affected eye	Fellow eye	*P*
0-1	21.03 ± 1.33	20.59 ± 1.21	0.243	—	—	—
1-2	21.43 ± 1.20	20.98 ± 0.74	**0.012** ^∗^	—	—	—
2–6	22.11 ± 1.72	21.84 ± 0.89	**0.048** ^∗^	43.69 ± 1.83	43.58 ± 1.52	0.547
6–12	23.48 ± 1.99	22.94 ± 1.40	**0.018** ^∗^	43.60 ± 1.57	43.61 ± 1.52	0.940
12–18	24.53 ± 1.93	23.31 ± 1.97	0.123	43.18 ± 0.43	43.22 ± 0.48	0.843
All children	22.34 ± 1.89	21.94 ± 1.27	**<0.001** ^∗^	43.61 ± 1.65	43.56 ± 1.46	0.621

^∗^Significantly different between the affected eye and fellow eye using paired *t*-tests. Results are presented as the mean ± standard deviation. AXL = axial length.

**Table 4:** Comparison of axial length and *K* value between the randomly selected eye and the contralateral eye in bilateral congenital cataract children.

Age (years)	AXL (mm)			*K* value (D)		
	Selected eye	Fellow eye	*P*	Selected eye	Fellow eye	*P*
0-1	19.54 ± 1.37	19.70 ± 1.42	0.127	—	—	—
1-2	20.86 ± 1.45	20.93 ± 1.46	0.406	—	—	—
2–6	21.90 ± 1.62	21.94 ± 1.67	0.542	43.49 ± 1.70	43.40 ± 1.94	0.561
6–12	22.45 ± 1.82	22.67 ± 2.01	0.080	44.06 ± 2.50	44.19 ± 2.39	0.109
12–18	24.79 ± 3.07	24.83 ± 2.96	0.731	43.21 ± 2.08	42.95 ± 2.29	0.238
All children	21.79 ± 2.01	21.89 ± 2.06	0.053	43.64 ± 2.03	43.60 ± 2.15	0.648

Results are presented as the mean ± standard deviation. AXL = axial length.
